# Obituary: Geoffrey Schild (1935–2017)

**DOI:** 10.1038/s41541-018-0045-9

**Published:** 2018-02-12

**Authors:** Philip Minor

**Affiliations:** National Institute for Biological Standards and Control, Potters Bar, EN6 3QG UK

Geoffrey Schild was one of the great influenza virologists of his generation, being largely responsible for the nomenclature system in use to categorise natural strains today and for the development of the method for assessing influenza vaccine potency that is one of the few fixed points in influenza vaccines. He also played a big part in the way the World Health Organisation dealt with influenza, particularly in the selection of vaccine strains, where he encouraged the involvement of the vaccine manufacturers in the annual meeting. His first degree was from Reading after which he spent time in Sheffield before joining the MRC National Institute for Medical Research in Mill Hill. He became the Head of the Division of Viral Products at the National Institute for Biological Standards and Control (NIBSC) in the late 1970s.

The NIBSC emerged from the MRC NIMR and was set up in 1976 as the laboratory arm of the National Biological Standards Board, a quango established to provide independent scientific advice on reference materials and other issues in the control of biological medicines. Typically, biological medicines are made in biological systems, such as cell culture, or by fractionation of starting materials, such as human blood. Initially they were complex and impure mixtures characterised by their biological activity rather than by their physico-chemical properties. Their control often depends on the availability of biologically active reference preparationsm and viral contamination is always an issue.

Viral vaccines are biological medicines and at the time that Geoffrey became Head of the Division of Viral Products, state-of-the-art academic methods had not really been applied to them. This was an issue for live polio vaccines, where every batch was tested in primates by methods based on those used by Albert Sabin in the 1950s for developing the vaccine initially. At the time, the methods were poorly designed and standardised; many animals provided no information and comparison of results was not possible. The situation became critical, when a batch passed by the Canadian Authorities failed, when tested in the UK. A second issue was the depletion of the viral seeds used to make the vaccine. No one really knew what they were measuring. Better characterisation was needed and Geoffrey set up a programme to address the matter, which extended to other aspects of virology relevant to biologicals including blood products.

He became the director of NIBSC in 1985, and at this time the use of recombinant DNA technology to produce biological medicines was in development, including the synthesis of insulin in bacteria and Hepatitis B surface antigen for vaccine production in yeast. Insulin had previously been prepared from porcine pancreas and Hepatitis B vaccines from the plasma of chronically infected patients. The use of expression techniques obviously makes production easier to control and remove hazards, such as unidentified infectious agents in the starting materials. The process of improving the quality of biologicals gained momentum in the 1980s and, under Geoffrey, the NIBSC played a major global role in developing the rules. The consequence is better characterised products of greater safety. He involved the NIBSC in a collaborative programme with the UK blood transfusion service to address the quality of blood and blood transfusion; part of that was to develop the working reagents so that the tests for blood-borne viruses such as HIV, Hepatitis B and Hepatitis C could be compared from laboratory to laboratory and the performance could be improved and monitored. The result is that transmission of viral infections by blood transfusion in the UK is now very rare. He played a major role in the introduction of standardisation for the detection of viruses in blood-derived products, such as clotting factors for the treatment of haemophilia, so that the results from manufacturers and regulators could be compared. Thus, product safety was improved without disruption of supply.

There is no doubt that his heart was with influenza, and the focus on this aspect of his career in other obituaries is reasonable, but his contribution to public health went far wider; he was the director of the UK AIDS-directed programme which punched above its weight globally, his interactions with WHO on all topics were extensive and his contributions were global in their effect. He was also the first chairman of the Working party on biotechnology of the EU, which is the forum in which the technical criteria for product quality were discussed and set. He was an energetic and enthusiastic man who believed passionately in science-led regulation. Some people found him over enthusiastic, others (me among them) found him just about right and a driver of events. He retired from the NIBSC in 2003. He is still missed.

